# The Metropolitan Asylums Board

**Published:** 1905-08-12

**Authors:** 


### The Hospital.

A Journal of

The Medical Sciences and Hospital Administration.

Vol. XXXVIII.—No. 985. SATURDAY, AUGUST 12, 1905.

### THE METROPOLITAN ASYLUMS BOARD.

The annual report of the Metropolitan Asylums Board for 1904, which has just been published, contains a very remarkable record of good work on behalf of some of the most helpless of the many suffering classes of the metropolis. With regard to infectious diseases the Board is able to report that the actual number notified of the cases with which it deals were about the same as in 1903. The results of treatment, as shown by the percentages of deaths and of recoveries, differ very little from those of the preceding year; and it is probable, now that the diphtheria mortality has been reduced to about ten per cent. on admissions, and to nil as regards cases treated by antitoxin on the first day of the disease, that future improvement is scarcely to be expected in any other direction than as a consequence of the earlier admissions which the policy of the Board seems likely to bring about. The more widely the character of the hospitals becomes known to the classes to which they minister the more likely are patients, and more especially the young, to be brought to them in good time, and to derive the whole of the benefits which science and experience are able to confer.

The report of the children's department, not for the first time, contains a very serious indictment of some of the metropolitan Boards of Guardians, on account of their systematic neglect to avail themselves, for the benefit of the children under their charge, of the advantages which the various institutions of the Asylums Board hold out. The Children's Committee point out that, although they have every reason to be satisfied with the results obtained at the Milfield Home, for early cases of pulmonary tuberculosis, and notwithstanding the clamour for the public provision of special treatment for consumptives, a considerable number of vacancies remain unfilled at the home. Beds are also available for the surgical treatment of tuberculosis, both at East Cliff House, Margate, and at St. Anne's Home, Herne Bay, and the Committee pronounce it to be doubtful whether all cases of ringworm and of ophthalmia are sent to the Board's schools, in which expert medical treatment is carried on side by side with education. The importance of these statements is accentuated in the report of the Visiting Ophthalmic Surgeon to the Board, Mr. E. Treacher Collins, F.R.C.S., who refers to the very unfavourable opinion of the curability of trachoma which is entertained by some ophthalmic surgeons, whose experience of treating it has been limited to the out-patient departments of hospitals, and adds that it is the more satisfactory to be able to report 82 cases of trachoma as discharged cured, and only seven cases as having returned with a relapse of the disease. He says also that many of the cases of trachoma are received at an early stage, when they are most amenable to treatment, and when a cure can be effected without the extensive scar formation in the conjunctiva which is a necessary consequence of advanced disease. The removal of the trachomatous follicles by operation has materially shortened the duration of treatment in a large number of cases, and the *x*-rays have been extensively employed with satisfactory results.

The case thus urged against the metropolitan guardians by the Committee is rendered still worse than it would at first sight appear by the fact that, under the financial arrangements now in operation, the whole cost of the institutions referred to is practically paid for by guardians in proportion to the rateable value of the parishes and Unions. Each board of guardians contributes the same amount to the institutions in question, whether they make use of them or not, and hence the withholding of suitable cases is so far from being a measure of economy that it actually entails additional expense on the Boards concerned, as they now pay for the maintenance of the cases which they might transfer, in addition to paying their full share of the cost of the homes or other institutions which are ready to receive them. We much fear that the whole story is but the repetition of an oft-told tale. Guardians are elected, in many of the parishes of the metropolis, not because they have any intention of discharging the duties of the office, but because they are local "political" busybodies, who must somehow or somewhere be in evidence. To do them justice, they are probably often too ignorant to be in the least degree capable of understanding the amount of mischief which may arise from their indifference and neglect, but their ignorance, unfortunately, has no tendency either to diminish the amount of this mischief or to mitigate its effects. The case is rendered worse by the fact that the complaint now made by the

Children's Committee has been made on several previous occasions, and it seems likely, unless the Press can succeed in arousing the attention of the ratepayers to the facts, that it may continue to be made indefinitely. For the present, at least, it appears that costly institutions are kept open at the expense of the rates, and are provided with every advantage and appliance that skill and knowledge can suggest. In the face of this fact, Boards of Guardians pay twice over for the cases which the institutions would receive, and at the same time deprive them of the advantages which have been provided for their benefit. It is impossible to imagine a more stupid or a more odious tyranny.

